# S193. EX VIVO SIGNATURE OF PSYCHOSIS AND TREATMENT RESPONSE IN PATIENT-DERIVED NEURONS

**DOI:** 10.1093/schbul/sby018.980

**Published:** 2018-04-01

**Authors:** Rakesh Karmacharya, Bradley Watmuff, Annie Kathuria, Bangyan Liu

**Affiliations:** Harvard University

## Abstract

**Background:**

Postmortem studies in schizophrenia show well-replicated neuronal differences in the prefrontal cortex (PFC), specifically showing lower dendritic spine density in upper-layer cortical pyramidal neurons. Animal models that recapitulate features of psychosis also show lower dendritic spine density and synapse number in the PFC, with a more pronounced effect in upper-layer cortical neurons. Furthermore, the decrease in dendritic spines and synapses in animal models have been shown to be reversible with antipsychotic treatment. Results from postmortem brains, animal models and in vitro rodent cultures provide a strong impetus to test the hypothesis that dendritic spine biology plays an important role in the biology of schizophrenia and in mediating the effects of antipsychotic medications.

**Methods:**

To extend these findings, we studied cortical neurons generated from subjects with schizophrenia. We reprogrammed induced pluripotent stem cells (iPSCs) from human subjects with schizophrenia and from matched healthy controls. We differentiated human iPSCs along the forebrain lineage to generate mature cortical neurons. We developed a robust experimental approach to delineate and quantify spines in the dendrites as well as methods to outline and measure the spines in order to classify the different spine types. We also developed methodology for functional characterization of individual neurons using calcium imaging in the cortical neuron cultures.

**Results:**

We found that cortical neurons generated from the iPSCs of schizophrenia patients had a lower density of dendritic spines when compared to cortical neurons generated from the iPSCs of healthy control subjects. We also delineated the different composition of spine types in cortical neurons from schizophrenia patients when compared to those from healthy control subjects. In cortical neurons from schizophrenia subjects, we found that clozapine exposure in vitro leads to a robust increase in dendritic spine density.

**Discussion:**

We found that cortical neurons from iPSCs of schizophrenia subjects recapitulate the dendritic spine differences reported in postmortem brains of schizophrenia subjects. Moreover, we found that human cortical neurons from schizophrenia subjects show increased dendritic spine density when exposed in vitro to clozapine. The ability to delineate cellular features related to disease biology in iPSC-derived neurons opens the door to understand the pathophysiology of schizophrenia and lay the foundations for the development of novel therapeutics.

